# Gut Dysbiosis and Its Role in the Anemia of Chronic Kidney Disease

**DOI:** 10.3390/toxins16110495

**Published:** 2024-11-17

**Authors:** Elisabet Coll, Secundino Cigarran, Jose Portolés, Aleix Cases

**Affiliations:** 1 Servei de Nefrologia, Fundacio Puigvert, 08025 Barcelona, Spain; 2 Anemia Working Group of the Spanish Society of Nephrology, 39008 Santander, Spain; portolesjpp@gmail.com (J.P.); acases@clinic.cat (A.C.); 3 Servicio de Nefrología, Hospital Ribera-Polusa, 27004 Lugo, Spain; secundino.cigarran@gmail.com; 4 Ressearch Net RICORS 2030 Instituto de Salud Carlos III ISCIII, 28029 Madrid, Spain; 5 Nephrology Department, Hospital Universitario Puerta de Hierro Majadahonda, 28222 Madrid, Spain; 6 Medicine Department, Facultad de Medicina, Research Institute Puerta de Hierro Segovia de Arana (IDIPHISA), Universidad Autónoma de Madrid, 28029 Madrid, Spain; 7 Nephrology Unit, Hospital Clinic, 08036 Barcelona, Spain

**Keywords:** gut dysbiosis, uremic toxins, anemia, ESA resistance, inflammation, chronic kidney disease

## Abstract

The gut dysbiosis present in chronic kidney disease (CKD) has been associated with anemia. Factors such as the accumulation of gut-derived uremic toxins, increased gut barrier permeability-induced inflammation, and a reduced intestinal production of short-chain fatty acids (SCFAs), all associated with changes in the intestinal microbiota composition in CKD, may lead to the development or worsening of anemia in renal patients. Understanding and addressing these mechanisms related to gut dysbiosis in CKD patients can help to delay the development of anemia and improve its control in this population. One approach is to avoid or reduce the use of drugs linked to gut dysbiosis in CKD, such as phosphate binders, oral iron supplementation, antibiotics, and others, unless they are indispensable. Another approach involves introducing dietary changes that promote a healthier microbiota and/or using prebiotics, probiotics, or symbiotics to improve gut dysbiosis in this setting. These measures can increase the presence of SCFA-producing saccharolytic bacteria and reduce proteolytic bacteria, thereby lowering the production of gut-derived uremic toxins and inflammation. By ameliorating CKD-related gut dysbiosis, these strategies can also improve the control of renal anemia and enhance the response to erythropoiesis-stimulating agents (ESAs) in ESA-resistant patients. In this review, we have explored the relationship between gut dysbiosis in CKD and renal anemia and propose feasible solutions, both those already known and potential future treatments.

## 1. Introduction

Anemia is a common condition in chronic kidney disease (CKD) and is associated with an increased risk of cardiovascular events, CKD progression, and mortality [1]. Furthermore, anemia has been associated with reduced health-related quality of life and increased healthcare resource utilization [2]. The prevalence of renal anemia increases as renal function declines, being almost universal in patients with end-stage renal disease (ESRD) [3,4]. The etiology of anemia in CKD is multifactorial, including an inadequate production of erythropoietin (EPO), as well as iron deficiency (absolute or functional), inflammation, increased levels of uremic toxins (most of them derived from the gut microbiota), reduced red blood cell lifespan, or nutritional deficiencies (vitamin B12 and folic acid), among others [5].

The gut microbiota is the aggregate of all microorganisms present in the human gastrointestinal tract. There is a crosstalk between the host and the microbiota, which is important for different key functions for the health of the host, such as modulating host physiology, digesting nutrients, producing vitamins and hormones (thus regulating the metabolism in the host), and inhibiting pathogen colonization while also preserving the gut barrier function and immunity [6]. In contrast, when the gut microbiota becomes dysbiotic, there is an excessive growth of pathogenic bacteria that produce byproducts that induce chronic immune activation and increase gut permeability, activating the intestinal–mucosa immune system, promoting the synthesis of proinflammatory cytokines and systemic inflammation [7].

Patients with CKD exhibit a significant gut dysbiosis with substantially reduced bacterial diversity and changes in the composition of their gut microbiota [8]. There is a decrease in the commensal saccharolytic bacteria that produce beneficial short-chain fatty acids (SCFAs) [9] and an increase in proteolytic bacteria that enhance the production of ammonia and gut-derived uremic toxins through the fermentation of urea and amino acids, increasing systemic inflammation [10]. SCFAs are bacterial fermentation products of difficult-to-digest carbohydrates, including dietary fiber (DF), such as acetate, propionate, or butyrate, and are crucial for maintaining the gut health. They are a source of energy for the enterocytes, improve glucose and lipid homeostasis, promote mucus production, and maintain gut barrier integrity and function. Moreover, they reduce intestinal pH, promoting commensal bacteria expansion, induce epigenetic modifications, and exert metabolic and anti-inflammatory effects [10], and may therefore modulate anemia in CKD through their anti-inflammatory effects. The estimated glomerular filtration rate (eGFR) shows a relationship with fecal butyric acid concentrations, which, in turn, correlates with the abundance of SCFA-producing bacteria, such as *Faecalibacterium prausnitzii* (renamed *F. duncaniae*), *Roseburia* spp., and *Butyricicoccus* spp. [11].

The leading causes involved in the gut dysbiosis of CKD patients are as follows:

1. Diet in CKD: The dietary restrictions of vegetables and fruits reduce DF intake. In this sense, DF from fruits, cereals, and vegetables and DF supplementation has been associated with improved glucose and lipid metabolism, body weight control, and improved intestinal barrier integrity through the production of SCFAs [12]. In a DF-rich diet, amino nitrogen is mainly incorporated into the bacterial biomass. At the same time, the production of SCFAs by saccharolytic fermentation predominates, keeping the intestinal pH low while reducing the proteolytic activity of bacteria that use SCFAs as an alternative source of energy. Associations between fecal and plasma levels of SCFAs, gut microbiota composition, and hemoglobin levels in ESRD hemodialysis (HD) anemic patients have been reported [13].

CKD is characterized by a decreased absorption of amino acids and a prolonged colonic transit time, which increases the amount of amino acids reaching the colon. This induces the expansion of proteolytic bacteria and could lead to an increase in the fermentation of aromatic amino acids into precursors of uremic toxins, such as p-cresol, indole, trimethylamine (TMA), or indole 3-acetic acid (IAA) [14].

2. The common prescription of some drugs in CKD, such as the following:

a. Antibiotics: antibiotics are commonly prescribed in CKD and their use reduces gut microbiota diversity and abundance, alters its metabolic activity, and produces a selection of antibiotic-resistant organisms, which can lead to antibiotic-associated diarrhea and recurrent *Clostridioides difficile* infections [15].

b. Oral iron: Oral iron is poorly absorbed in the duodenum, and the unabsorbed fraction reaches the colon, where it is involved in Fenton and Haber–Weiss reactions, which damage the intestinal structure [16]. Oral iron supplementation also alters the gut microbiota, while the composition of this microbiota modulates iron absorption [16]; therefore, iron and gut microbiota are in a complex and bidirectional relationship [17].

c. Phosphate binders: Some phosphate binders may impair the absorption of critical nutrients (e.g., vitamins and minerals), impact gut barrier integrity, and alter the gut microbiome [18]. In this sense, sevelamer use has been associated with increased uremic toxin levels and a decreased vitamin K status, suggesting a negative effect on gut bacteria metabolism [19] in some studies, but not all [18].

d. Potassium binders, especially polystyrene sulphonate, reduce colonic transit time, which may negatively impact on gut microbiota and potentially on gut-derived uremic toxins [20].

3. The increase in serum urea levels favors an influx of urea and uric acid into the intestinal lumen via the enterohepatic cycle, which significantly increases the number of bacterial species that express urease, uricase, and enzymes that generate indole (the precursor of indoxyl sulfate [IS]) and p-cresol (two well-known uremic toxins), while decreasing butyrate-producing species [21]. Bacterial urease hydrolyses urea into ammonia in the colon, which is transformed to ammonium hydroxide that increases the intestinal pH, promotes deleterious changes in the gut microbiota, and increases gut permeability through the depletion of tight junction proteins in the intestinal wall, which together with the decrease in butyrate synthesis favors systemic inflammation [22]. Altered gut barrier permeability allows macrophages to infiltrate and produce inflammatory cytokines that cause a local inflammatory response and enables the translocation of lipopolysaccharides (LPSs) and other bacteria-derived products into systemic circulation, further enhancing systemic inflammation [22]. This loss of intestinal barrier integrity and increased permeability is known as “Leaky gut” [7].

4. Gut wall edema and HD-induced ischemia are known to enhance gut permeability, resulting in endotoxemia and even bacterial translocation, consequently favoring systemic inflammation. In this sense, the fluid overload common in CKD patients favors gut wall edema, which increases intestinal barrier permeability. Moreover, hypotensive episodes secondary to excessive ultrafiltration during HD could promote intestinal ischemia and endotoxin translocation [22]. In fact, fluid overload in CKD has been associated with anemia [23,24].

The gut microbiota plays an important role in hematopoiesis [25]. Studies performed in germ-free mice demonstrated that these animals have smaller hematopoietic stem and progenitor cell populations [26]. Moreover, antibiotic treatment induces anemia and reduces hematopoiesis by depleting intestinal microbiota [27].

Germ-free mice show diminished intestinal hypoxia inducible factor (HIF) expression, while the SCFA butyrate increases oxygen consumption, which preserves HIF expression and gut barrier function. Antibiotic-mediated microbiota depletion reduces colonic butyrate and HIF expression, both restored by butyrate supplementation. The effects of butyrate are lost in cells lacking HIF, linking butyrate metabolism to stabilized HIF and the maintenance of gut barrier function [28]. Finally, as discussed later, gut dysbiosis in CKD and the consequent increased circulating levels of gut-derived uremic toxins play a role in CKD-associated anemia.

**Figure 1:**
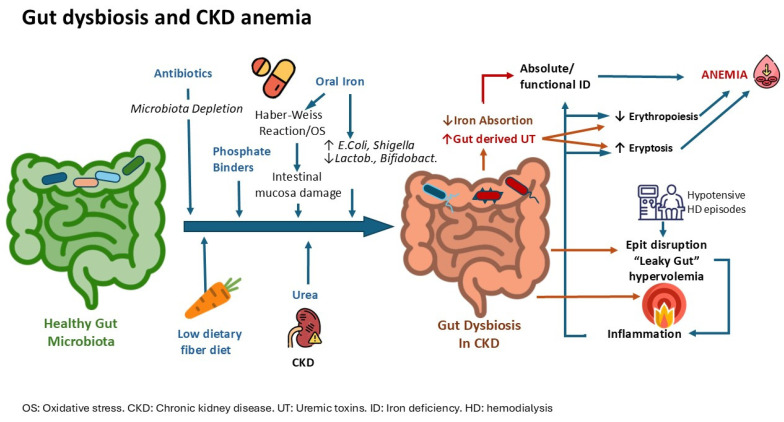
Causes and consequences of gut dysbiosis in CKD patients and its role in anemia.

**Figure 2:**
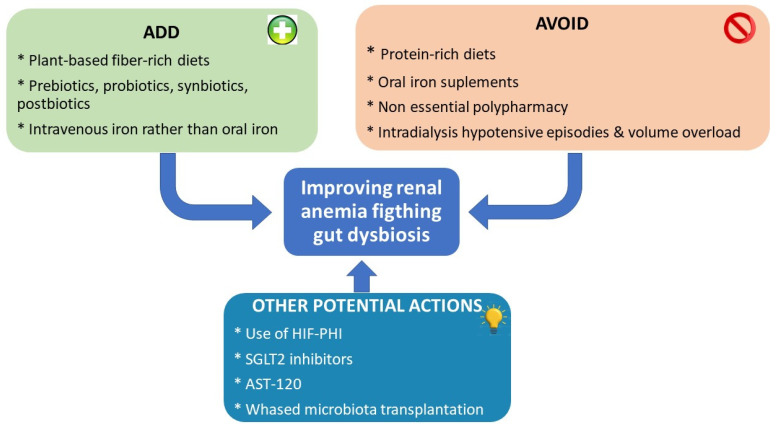
Therapeutic approaches to prevent/correct renal anemia related to gut dysbiosis.

This review aims to highlight the role of the gut microbiota in CKD-related anemia by describing the interactions, causes, and consequences (Figure 1) while also discussing the possible therapeutic approaches (Figure 2).

## 2. Gut-Derived Uremic Toxins and Anemia in CKD

When CKD progresses, gut-derived uremic toxins accumulate in the blood due to their impaired renal excretion and have been associated with several CKD-related complications, like atherosclerosis, cardiovascular disease, peripheral artery disease, increased mortality, or even anemia, among others [29]. Some gut-derived uremic toxins favor anemia by reducing EPO synthesis and the erythropoietic response by inhibiting HIF activation and enhancing eryptosis (the premature stress-induced programed death of red blood cells). In fact, the addition of uremic serum from HD patients enhances eryptotic markers on red blood cells (RBCs) from healthy controls, while inhibition of the organic anion transporter 2 with ketoprofen (that inhibits the uptake of uremic toxins by RBCs) and the incubation of HD patients’ RBCs with serum from healthy controls reverted eryptosis, suggesting a direct role of uremic toxins in RBC eryptosis in CKD [30].

Among the gut-derived uremic toxins, IS is produced through the fermentation of tryptophan by colonic bacteria that produce indole, which is absorbed and then conjugated to IS by the liver [14]. IS accumulates in CKD and contributes to anemia through several mechanisms.

IS induces eryptosis, characterized by erythrocyte shrinkage due to extracellular Ca^2+^ entry and phosphatidylserine exposure to the outer RBC membrane leaflet [31,32], thus reducing RBC lifespan. Other uremic toxins, such as urea, p-cresol, acrolein, or IAA, have also shown to enhance eryptosis in CKD [33,34,35].

IS also inhibits EPO synthesis by reducing nuclear HIF accumulation and its transcriptional activity in vitro [36]. Furthermore, the administration of indole (the precursor of IS) suppresses the hypoxic induction of EPO mRNA and protein in vivo [36]. These findings were confirmed in other studies showing that IS reduced the hypoxic induction of HIF-1 target genes by inhibiting its transcriptional coactivators [37]. IS is a potent agonist of the aryl hydrocarbon receptor (AhR) [38], which upon binding translocates to the nucleus and forms a heterodimer with the AhR nuclear translocator (ARNT) (or HIF-β). IS, at concentrations similar to the serum levels found in CKD patients, suppressed hypoxia-induced EPO mRNA expression and the transcriptional activation of HIF through AhR activation. The blockade of AhR abolished the IS-induced suppression of HIF activation in HepG2 cells. Furthermore, IS suppressed the nuclear accumulation of the HIF-α-ARNT complex, while the AhR-ARNT complex increased in the nucleus, suggesting a competitive interaction between AhR, HIF-α, and ARNT (HIF-β) in the inhibitory mechanism of the EPO synthesis of IS [38]. In the same study, the oral administration of indole in rats inhibited a bleeding-induced elevation of renal EPO mRNA expression and plasma EPO concentration and strongly induced AhR activation in the liver and renal cortex tissues. The inhibitory role of IS in the EPO synthesis in CKD was confirmed in a study with sulfotransferase 1a1-deficient mice, the enzyme that metabolizes indole to IS in the liver. In this study, EPO expression was reduced after unilateral ureteral obstruction in control animals, but in *Sult1a1*-KO mice, EPO mRNA expression improved considerably, associated with a reduction in IS levels, as well as kidney fibrosis and inflammation [39]. Indoxyl glucuronide, but not other gut-derived uremic toxins, such as p-cresyl sulfate (PCS), phenyl sulfate, or IAA, inhibited hypoxia mimetic-induced EPO mRNA expression and inhibited the transcriptional activation of HIF through the activation of AhR [40].

Uremic serum inhibits erythropoiesis [41], and this effect is partially reverted by hemodiafiltration with endogenous reinfusion that uses an adsorbent cartridge that allows for a better removal of uremic toxins [42], suggesting a negative role of uremic toxins on erythropoiesis. IS inhibits erythropoiesis, inducing apoptosis in the UT7/EPO cell line and blockage at the burst-forming unit-erythroid (BFU-E) stage in vivo and in vitro [43] and there is a shift in these precursors to a megakaryocytic phenotype. Furthermore, IS downregulates the expression of erythropoietic-related genes, such as GATA-1, EPO-receptor (EPO-R), or ẞ-globin, and downregulates the EPO-EPO-R signaling pathway. Additionally, IS reduces proliferation, impairs erythrocyte differentiation capacity, and promotes cell apoptosis and senescence during erythropoietic differentiation [43,44]. IS also promotes ESA resistance and endothelial dysfunction by inhibiting the EPO-induced tyrosine phosphorylation of EPO-R and Akt phosphorylation (an intracellular signal transduction pathway that promotes the survival and growth of erythroblasts) and reduces the expression of thrombospondin-1, an erythroid-stimulating factor [45].

There is a significant negative correlation between IS and EPO levels in CKD patients. Moreover, the EPO gene and protein expression were reduced in CKD rats, which was significantly reversed by lowering serum IS with the oral adsorbent AST-120 [46], which decreases its serum levels by adsorbing indole from the gut and promoting its excretion through the feces [47]. However, no relationship has been found between the serum levels of uremic toxins, including IS, and hemoglobin (Hb) levels or other anemia parameters in CKD patients in most studies [48,49,50], but not in all [51,52]; the association was lost in the multivariate analysis, while in one negative study, only in the subset of non-anuric peritoneal dialysis patients a relationship was found between IS and Hb levels [48].

IS also induces hepcidin synthesis in a dose-dependent manner in HepG2 cells through the AhR and oxidative stress pathways. In vivo experiments with adenine-induced CKD mice showed increased hepatic and plasma hepcidin levels, reduced serum iron levels, and increased serum ferritin and splenic iron, as well as a reduced duodenal expression of ferroportin, suggesting that IS also impairs iron absorption and utilization. The reduction in IS levels with AST-120 in CKD mice blunted the increase in hepcidin, improved erythropoiesis, and reversed most of the previous changes [53].

Both food but also gut microbiota are sources of polyamines [54], uremic toxins which have been reported to reduce the proliferation and maturation of erythroid precursor cells (CFU-E) by acting as inhibitors of erythropoiesis in ESRD patients [55].

Asymmetric dimethyl arginine (ADMA) is a uremic toxin that accumulates in CKD, and it is associated with an increased cardiovascular risk. Several gut bacteria have the potential to produce ADMA [10], despite there being no studies addressing the relationship of endogenous ADMA synthesis/metabolism and the role of gut microbiota. Increased ADMA levels have been associated with increased NLR family pyrin-domain-containing 3 (NLRP3) inflammasome expression in the serum and ileum, increased Toll-like receptor 4 (TLR4), and decreased expression of tight junction proteins in the ileum, together with changes in the gut microbiota composition [56], effects that were partially reverted by the polyphenol resveratrol. Finally, increased erythrocyte ADMA accumulation contributes to an impaired response to EPO in CKD patients and mice with advanced CKD via the suppression of EPO-R expression [57].

## 3. Inflammation, Gut Microbiota, Uremic Toxins, and Anemia

CKD is a chronic inflammatory state, and inflammation has been involved as one of the mechanisms of anemia in this setting [5,58]. As previously mentioned, gut dysbiosis in CKD contributes to immune dysfunction and inflammation [8,59], and it is associated with increased gut permeability [7], which activates the nuclear factor-kappa β (NF-κβ) pathway with the chronic production of proinflammatory cytokines, leading to systemic inflammation. There is increasing evidence that the intestinal barrier dysfunction contributes to systemic inflammation in CKD patients [22].

Several studies have shown increased gut permeability to large-molecular-weight polyethylene glycols in CKD [60]. In addition, histological studies have revealed the presence of chronic inflammation throughout the gastrointestinal tract in HD patients [61], and several studies have revealed the presence of endotoxemia, in the absence of infection, in uremic patients and its relationship with systemic inflammation [59,62,63,64]. The translocation of endotoxins and other bacteria-derived end products can stimulate the immune system cells, especially macrophages and endothelial cells, to become activated and secrete a wide variety of proinflammatory cytokines [7,65].

Gut-derived uremic toxins also enhance systemic inflammation. P-cresol may have deleterious effects on intestinal barrier function [66,67]. Trimethylamine-N-oxide (TMAO), another gut-derived uremic toxin, is associated with the marker of gut permeability zonulin and LPSs and inflammatory biomarkers in type 2 diabetic patients with advanced CKD [68]. Further, TMAO may favor gut inflammation by reducing autophagy and activating NLRP3 inflammasome in fetal human colon cells [69]. IS also induces inflammation and oxidative stress [70] and downregulates the expression of the antioxidant system nuclear factor (erythroid-derived 2)-like 2 (Nrf2) and its related genes [71]. In this sense, there is a relationship between markers of inflammation and the serum levels of uremic toxins [72]. IAA levels were positively associated with markers of inflammation and oxidative stress, C-reactive protein, and malondialdehyde, respectively. In cultured human endothelial cells, IAA activated the proinflammatory AhR/p38MAPK/NF-κβ pathway that induced the proinflammatory enzyme cyclooxygenase-2 (COX-2) and increased the production of endothelial reactive oxygen species [73]. Similar proinflammatory effects have been described with TMAO [74], and a relationship between TMAO and inflammatory markers in CKD has also been reported [75].

Bacteria-derived LPS infusion reduces EPO expression through activation of the NF-κβ pathway [76]. In inflammatory states, the EPO secretion in response to anemia is blunted [77,78]. The addition of IL-1α, IL-1β, or TNFα to HepG2 cultures resulted in a decrease of up to 60% in baseline EPO production [78]. In addition, these inflammatory cytokines reduced hypoxia-induced EPO production in Hep3B cells [79] and reduced EPO synthesis by suppressing HIF activation [80,81,82,83]. In fact, HIF-1α and NF-κβ require the same coactivator p300 to stimulate their transcription genes; thus, both transcription factors compete for this coactivator, and the simultaneous activation of both will be mutually exclusive, while HIF modulates NF-κβ activity [84].

In CKD, renal EPO-producing cells (REPCs) are transformed into myofibroblasts [85], losing the ability to produce EPO, a reversible phenomenon after elimination of the injury [86]. Transforming growth factor-β (TGF-β) and TNF-α facilitates the conversion of REPCs into myofibroblast cells by activating SMAD and NF-κβ transcription factors, which ultimately reduces EPO synthesis in REPCs [81].

Inflammatory cytokines also directly suppress erythropoiesis, especially at the CFU stage of development, an effect that is partially improved by EPO [77]. Inflammation also promotes a shift in hematopoietic stem cells to myeloid differentiation via NF-κβ and the transcription factor PU.1, which promotes myelopoiesis and lymphopoiesis at the expense of erythropoiesis and shifting the multipotent progenitor compartment to a megakaryopoietic phenotype rather than an erythropoietic one [84,87]. TNF-α inhibits the differentiation and proliferation of erythroid progenitor cells [88,89] and causes erythrophagocytosis and dyserythropoiesis [90]. Furthermore, the bone marrow response to EPO is blunted in proinflammatory states [91]. Increased levels of inflammatory cytokines, such as interferon-γ, IL-1β, and TNF-α, commonly seen in CKD, may lead to a decreased sensitivity of erythroid progenitors to EPO [92,93,94,95].

Inflammation is also associated with erythrophagocytosis (the phagocytosis of senescent RBCs) [96], as well as enhanced eryptosis [33,97], thus reducing RBC lifespan [98].

Clonal hematopoiesis of indeterminate potential (CHIP) is an age-associated hematologic disorder that is associated with increased morbidity and mortality, both in the general population and CKD patients [99]. There is a bidirectional relationship between CHIP and inflammation. Myeloid cells carrying CHIP mutations produce high levels of inflammatory cytokines, while proinflammatory cytokines promote clonal expansion [100]. The presence of CHIP has been associated with lower Hb levels and a higher mean corpuscular volume and ferritin levels in CKD patients, suggesting that it may be a contributing factor in anemia in CKD [101].

Inflammatory cytokines also impair iron metabolism. TNF-α induces hypoferremia by inhibiting iron release from macrophages [102] and increasing the transcriptional induction of ferritin in several cell lines [103]. TNF-α also inhibits iron uptake in erythroid precursors. Recent in vitro and animal studies have shown that TNF-α also directly inhibits intestinal iron absorption [104], independently of hepcidin production [104]. In addition, TNF-α inhibited the ferroportin-1 and hemojuvelin mRNA formation in HepG2 cells. Ref. [105] TNF-α and IL-1 increase the number of transferrin receptors in cultured human fibroblasts [106]. Conversely, erythroid precursors isolated from patients with rheumatoid arthritis (RA) and anemia of chronic inflammation express significantly fewer transferrin receptors than normal controls or non-anemic patients with RA [107]. These conditions favor incorporating iron into storage tissues rather than bone marrow erythroblasts. Finally, hepcidin (whose levels are increased in the presence of inflammation or CKD) inhibits erythropoiesis [108].

High-mobility group box-1 (HMGB1) acts as a damage-associated molecular pattern (DAMP) or alarmin to activate the immune response, it is a key inflammatory mediator [109] and has an association with many diseases involving inflammation, including CKD. HMGB1 has been found to influence the biological function of the intestinal mucosa [110], and it is associated with anemia in animal models of sepsis. In one preclinical model of sepsis, HMGB1 induced anemia, led to reduced expansion, especially at the CFU stage, increased the death of EPO-sensitive erythroid precursors in human models of erythropoiesis, and significantly attenuated EPO-mediated phosphorylation of the Janus kinase 2/STAT5 and mTOR signaling pathways. Furthermore, surface plasmon resonance studies show the capacity of HMGB1 to interfere with the binding between EPO and the EPOR. The administration of a monoclonal anti-HMGB1 antibody after sepsis onset in mice partially restored EPO [111] signaling in vivo.

The gut microvascular endothelium plays a central role in mucosal immunity. Sodium butyrate modulates the mucosal innate immune response towards LPSs through effects on microvascular endothelial function, since it increased ICAM-1, while it inhibited IL-6 and COX-2 mRNA expression and protein and decreased prostaglandin E2 production in response to LPSs [112].

SCFAs maintain gut epithelial barrier function [113] and reduce gut and systemic inflammation [113]. Furthermore, SCFAs attenuate inflammatory processes triggered by proinflammatory cytokines, such as TNF-α [114]. Therefore, the enhanced inflammation and increased uremic toxins levels, together with the decrease in SCFAs associated with gut dysbiosis in CKD, promotes inflammation that may contribute to anemia in CKD (Table 1).

## 4. Gut Microbiota and ESA Responsiveness in CKD Patients

ESA resistance in anemic CKD patients is relatively common, especially in dialysis patients. The most common causes of ESA resistance include iron deficiency, inflammation, secondary hyperparathyroidism, inadequate dialysis, and concomitant medications [115].

In this sense, changes in the gut microbiota composition can explain some cases of ESA hyporesponsiveness. In a cross-sectional study in ESA-treated HD patients, nine bacterial genera could predict ESA hyporesponsiveness (Neisseria, Streptococcus, Porphyromonas, Fusobacterium, Prevotella_7, Rothia, Leptotrichia, Prevotella, and Actynomices). In contrast, five bacterial genera were associated with good responses to ESA (Bifidobacterium, Faecalibacterium, Citrobacter, Escherichia-Shigella, and Bacteroides) [116]. Among these genera, an increase in the probiotic *Bifidobacteria* had a good predictive value (AUC = 0.77), suggesting that supplementation with probiotics may be an adjuvant therapy for renal anemia and ESA hyporesponsiveness in ESRD. A functional analysis showed that most butyrate synthesis-related enzymes were significantly enriched among the good responders, suggesting that these patients may increase gut butyrate production, which may be relevant due to its anti-anemic effects. Furthermore, in a randomized controlled trial (RCT) in NDD-CKD anemic patients, the addition of the activated charcoal oral adsorbent AST-120 to pegylated EPO (CERA) increased Hb levels more than CERA alone; more patients achieved a Hb > 11 g/dL in the combination group and the doses of CERA were lower with the combination. These effects were associated with decreases in IS and other uremic toxins [117], thus suggesting that AST-120 improves the response to ESA by decreasing IS levels. No changes in serum ferritin and TSAT were observed in both groups in this study.

## 5. Iron and Its Connection with Gut Microbiota and Host

Iron is an essential trace element for almost all aerobic organisms and most bacteria. Iron homeostasis is tightly controlled in mammals, especially in the duodenum, where iron is absorbed. Iron is necessary for several critical physiological and cellular activities, like mitochondrial respiration, oxygen transport and storage, DNA synthesis, antioxidant defense, and metabolic pathways [5]. Iron is also essential for bacteria, as it functions as a co-factor in iron-containing proteins in redox reactions, metabolic pathways, and electron transport chain mechanisms [118]. Gut bacteria populations compete with the host for iron [17]. Microbes acquire iron by producing siderophores, which are small molecules that chelate and internalize iron. Siderophores play a major role in microbial physiology and virulence, and they can modulate interbacterial competition and host cellular pathways [119]. In the presence of iron deficiency, gut microbiota produces metabolites that inhibit HIF-2α expression and increases ferritin levels, which reduces intestinal iron absorption [17]. Moreover, iron deficiency or excess may influence gut microbiota; however, the mechanism/s is/are still unknown and deserves further studies [120]. Therefore, maintaining a balanced iron level is essential for preserving intestinal and overall health.

## 6. Iron Supplementation and Gut Microbiota

CKD patients frequently require oral iron treatment to treat iron deficiency anemia (IDA). Nevertheless, oral iron is poorly absorbed (up to 15–20%), and the rest remains in the intestinal lumen to be utilized by the microbiota, mainly in the colon. Supplementation may be deleterious for the gastrointestinal system through (1) the generation of free radicals via iron-induced redox cycling in the gut lumen and the mucosal surface that may promote inflammation and (2) by promoting changes in the gut microbiota composition or metabolism, since it increases luminal iron content, which is used by some iron affinity pathogens that are harmful to the patients [16]. The colonic iron favors the growth of pathogenic bacteria and decreases the *Bifidobacteria* population, which is associated with intestinal inflammation and diarrhea [118].

In a prospective study that evaluated the effects of iron supplementation on gut microbiota, 28 HD patients were randomized to receive either oral iron (200 mg of ferrous succinate once a day) or intravenous iron (100 mg of iron sucrose three times a week). Oral iron reduced α-diversity, decreased the relative abundance of *Firmicutes*, significantly increased the abundance of *Bacteroidetes* at the phylum level, and reduced the abundance of SCFA-producing bacteria compared with the intravenous iron group. However, the Lactobacillus genus was more abundant in patients on oral iron therapy. According to metagenome function prediction analysis, oral iron increased the amino acid metabolism-related genes, changes that may favor the production of gut-derived uremic toxins, suggesting that IV iron may be a better alternative for CKD patients [121].

The effect of the iron-containing phosphate binder sucroferric oxyhydroxide (SFO) on uremic toxins and the gut microbiome was evaluated in 18 HD patients after three months of administration and compared to a control group of 20 HD patients without SFO. Serum IS and PCS increased in the group treated with SFO but did not change in the control group despite no significant changes in the gastrointestinal microbiome were observed [122]. The lack of changes in the gut microbiota with SFO were confirmed in two other studies [123,124].

Ferric citrate, another phosphate binder, has been approved for treating hyperphosphatemia and for IDA in CKD patients. One study evaluated the differences in gut microbiota after treatment with two phosphate binders (ferric citrate vs. calcium carbonate) in HD patients. Ferric citrate treatment enhanced gut microbiome diversity with an increase in the Bacteroidetes species and a reduction in the Firmicutes phylum. Taxa of the genera Ruminococcaceae UCG-004, Flavonifractor, and Cronobacter were increased in the group of patients that received ferric citrate [125]. The functional characterization of the microbiome revealed that significantly enriched pathways in ferric citrate users included cholesterol biosynthesis, clavaminate biosynthesis, or enterotoxigenic *Escherichia coli* and Vibrio cholera pathogenicity signatures. Studies on the effects on gut microbiota, gut barrier function, and inflammation using ferric citrate in CKD rats show discordant results [126,127,128].

## 7. Dietary Patterns, Gut Microbiota, and Anemia in CKD Patients

Healthier diets such as vegetarian/vegan or Mediterranean diets (as opposed to a Western diet) may be beneficial for CKD patients [129,130], since they can improve gut dysbiosis in several ways as follows:

1. A diet rich in fiber promotes an expansion of saccharolytic bacteria producing SCFAs and decreasing proteolytic bacteria species [129], subsequently reducing the formation of gut-derived uremic toxins [131]. In CKD patients, the levels of SCFAs inversely correlate with renal function [11].
2. The increased production of SCFAs provides energy to colonocytes and the gut microbiota, allowing amino acids to be incorporated into the colonic bacterial proteins and be excreted in feces instead of being fermented to uremic toxins [132]. In addition, SCFAs also aid in sustaining the functionality and integrity of the intestinal barrier, preserve the luminal pH, impede the growth of pathogens, and favor intestinal motility [12].
3. By decreasing the intestinal transit time and consequently the time for proteolytic degradation, there is a reduction in the production of bacterial metabolites, such as ammonia, phenols, indoles, and amines, which ameliorates the composition of the dysbiotic microbiota and increases the excretion of human and bacterial byproducts, consequently decreasing the production/absorption of uremic toxins [133]. A prolonged intestinal transit time promotes the conversion of amino acids into uremic wastes through microbial fermentation [20].

A recent RCT found improved Hb levels in HD patients with soluble DF supplementation, associated with changes in the gut microbiota and SCFAs [13]. DF may also reduce the production of uremic toxins [134] and their producing bacteria in the gut microbiota. However, the potential beneficial effects of DF on hemoglobin or iron metabolism markers have not been demonstrated in a systematic review [135]. Furthermore, plant-based diets can reduce markers of inflammation in CKD [136,137], but the New Nordic Renal Diet (a Nordic equivalent to the Mediterranean diet), a plant-based renal diet rich in DF, failed to reduce CRP [138] or increase Hb levels, despite decreasing the urinary excretion of IS and PCS [138].

Low- or very low-protein diets can also improve gut dysbiosis and reduce gut-derived uremic toxins and gut permeability in CKD patients [139,140]. However, in a recent meta-analysis on the effect of low protein diets, despite improvements in gut microbiota in CKD, there was heterogeneity in the impact on IS or PCS and there were no changes in Hb levels [141]. Similarly, no differences in Hb changes were seen in a study in CKD patients comparing a low-protein diet supplemented with ketoanalogs and the control group [142].

## 8. Prebiotics, Probiotics, Symbiotics, Gut Microbiota, and Renal Anemia

Prebiotics are nondigestible food components that confer health benefits by modulating the host’s gut microbiota. Prebiotics mainly include plant-derived products, including complex carbohydrates such as inulin, fructo-oligosaccharides, arabinoxylan-oligosaccharides, resistant starch, guar gum, etc., as well galacto-oligosaccharides and some non-carbohydrates, such as cocoa-derived flavanols or resveratrol.

Some studies showed an increase in total SCFAs (butyrate, acetate, and propionate) using arabinoxylan oligosaccharides at high doses (7.5 g per day), and similar results were shown if oligosaccharides were administered through fiber-enriched food [143,144]. Resistant starch supplementation reduced levels of IS or IL-6 in HD patients vs. placebos, although no effects on Hb levels were reported [145,146].

A previously mentioned RCT evaluated the effects of soluble DF on renal anemia as a primary endpoint. In total, 162 HD patients were randomly assigned into the DF group (10 g of DF mixture composed of galactomannan, resistant dextrin, fructo-oligosaccharides, and starch) and a control group. After 8 weeks, patients treated with DF had increased Hb levels by an average above 20%. An increase in iron and ferritin levels but no changes in serum hepcidin, soluble transferrin receptor, or ESA doses were observed. Moreover, in the intervention group, there was an increase in Bifidobacterium adolescentis, Lactobacillus, and Lactobacillaceae, as well as an increase in serum SCFAs (especially butyric acid). Most of these changes were positively associated with Hb levels and negatively associated with EPO dosage. Furthermore, butyric acid levels correlated with Lactobacillus and Lactobacillaceae. These results strengthen the hypothesis that the increase in Hb levels by DF in HD patients could be related to increased butyric acid secondary to changes in gut microbiota [13]. However, in another RCT using a prebiotic (12 g/day of fructo-oligosaccharide for three months) in non-diabetic CKD patients (n = 46), no changes in Hb levels (baseline Hb 12.7 g/dl) or IS were seen, despite reductions in IL-6 levels [147].

The World Health Organization defines probiotics as live strains of strictly selected microorganisms that when administered in adequate amounts confer a health benefit to the host. The most frequently administered probiotics are Bifidobacteria longum, B. bifidum, Lactobacillus acidophilus, *L. casei*, *L. sakei*, *L. reuteri*, and *Streptococcus thermophilus*. Bifidobacteria produce some vitamins and also SCFAs [148]. Symbiotics are described as a combination of synergistically acting probiotics and prebiotics.

The effect of probiotics was tested in 75 HD patients who were randomly assigned to receive symbiotics (n = 23) (15 g of prebiotics +5 g of probiotic containing Lactobacillus acidophilus, Bifidobacterium bifidum, B. lactis, and B. longus), probiotics (n = 23), or a placebo (n = 19). After 12 weeks, mean Hb levels showed a significantly increased trend in patients supplemented with the probiotic or symbiotic formulations compared to the placebo group [149].

In another RCT that evaluated the efficacy of probiotics on renal anemia in HD patients, eighteen patients received supplementation with probiotics for three months, whereas 18 patients received a placebo. Probiotic supplementation decreased Hb fluctuations but did not significantly increase Hb levels [150]. Other studies evaluating the use of probiotics in CKD patients failed to find significant effects on Hb levels [151,152]. Similarly, in a meta-analysis on the use of probiotics in CKD, no changes in Hb levels and CRP and increases in IL-6 among the probiotic users were reported [153], questioning their benefits for anemia in CKD. Similarly, in a study with a symbiotic in CKD patients, no significant changes in IS or proinflammatory cytokines were observed vs. a placebo, but no data on Hb changes were reported [154].

Therefore, despite the potential benefits of prebiotics, probiotics, or symbiotics in improving anemia in CKD, there is not a clear answer from the clinical studies due to the lack of studies specifically addressing this outcome, the low sample size in most studies, and the short time of follow-up in most of them.

Postbiotics are defined as a “preparation of inanimate microorganisms and their components that confers health benefits to the host”. Acetate, a SCFA, could directly stimulate erythropoiesis through a HIF-2-related pathway [155]. Sodium butyrate, a histone deacetylase inhibitor, can reactivate fetal hemoglobin, stimulating the proliferation of RBCs and has been considered as a therapy for sickle cell anemia and beta-thalassemia [156,157]. Further, the SCFAs propionate, butyric acid, and their related producing bacteria are effective against IDA [158]. This suggests that SCFAs, whose production is reduced in CKD, and the gut microbiota may impact anemia and iron parameters through several mechanisms. In this sense, the administration of oral sodium propionate to 20 HD patients for 12 weeks results in a decline in inflammatory parameters, a reduction in oxidative stress, a reduction in ferritin, and an increase in the transferrin saturation index, as well as a decrease in the serum levels of the uremic toxins IS and PCS [159].

Lactic acid is considered an intermediate substance produced by gut microorganisms and serves as a source of SCFAs [160], particularly butyrate. Lee et al. showed that microbiota-derived lactate stimulates stem cell factor (SCF) secretion by LepR+ bone marrow mesenchymal stromal cells and subsequently activates hematopoiesis and erythropoiesis in a Gpr81-dependent (lactate receptor) manner, but studies on anemia in CKD are lacking.

Some polyphenols can also modulate gut microbiota and reduce inflammation. In this sense, resveratrol improves gut barrier function and gut microbiota, and reduces the hepatic synthesis of IS, and likely may improve anemia in CKD [161]. Urolithin A, a microbial metabolite of polyphenols, also improves epithelial barrier function [162]. Curcumin is also associated with reduced inflammation and oxidative stress in CKD patients. However, despite the beneficial effects on oxidative stress and inflammation in dialysis patients [163,164], no beneficial effect on anemia was observed [163].

With respect to the type of HD, high-volume hemodiafiltration (HV-HDF) has shown better removal of small- and middle-molecule solutes and a higher reduction in inflammation and oxidative stress than with high-flux HD. However, the beneficial effect on Hb, ESA resistance, or iron parameters is inconsistent. While some small RCTs suggested a beneficial effect on the ESA resistance index (ERI) or hepcidin levels [165,166,167], large RCTs failed to find a benefit on Hb levels or ERI [168,169,170]; similarly, in the recent CONVINCE trial, no differences in Hb or CRP were seen between the two groups during the follow-up [171], although no data on ERI has been reported so far. This may be due to the limited clearance of protein-bound uremic toxins, such as IS, with HV-HDF vs. high-flux HD [172,173,174].

## 9. Fecal Microbiota Transplantation and Anemia of Chronic Disease

Patients with anemia of chronic diseases (ACD) have altered microbial fecal profiles [175]. Fecal microbiota transplantation (FMT) from healthy donors is an effective treatment to restore the balance of intestinal microbiota [176]. Nevertheless, its use in clinical practice is restricted because of significant adverse events, such as infections and deaths [177]. In this sense, washed microbiota transplantation (WMT) is an advancement in FMT, since it uses an automated process for purification and washing, reducing the adverse effects of FMT [178].

Recently, Zhong et al. evaluated the efficacy of WMT on hematological parameters in patients with ACD and analyzed changes in gut microbiota (n = 13, only three with anemia in CKD). WMT significantly improved ACD, restoring the normal Hb levels in 27.02%, 27.78%, and 36.37% after the first, second, and third interventions, respectively. Moreover, WMT produced changes in the gut microbiota composition, restoring the butyrate-producing bacteria Lachnospiraceae NK4A136 group and Butyricicoccus, which were decreased in patients with anemia and positively correlated with Hb levels [179].

## 10. Roxadustat and Gut Microbiota

HIF-1α has been shown to maintain the integrity of the intestinal epithelial barrier and to improve the survival of intestinal microorganisms [180]. HIF-2α regulates intestinal iron transporters mediated by the gut microbiota. Intestinal microorganisms and their metabolites may inhibit HIF-2α under conditions of iron deficiency [17]. Zhao et al. evaluated if the effects of Roxadustat (an inhibitor of the HIF-prolyl-hydroxylase) when administered to HD patients with ESA resistance were mediated by modulating the intestinal microbiota. Thirty HD patients with ESA hyporesponsiveness were evaluated before and after Roxadustat administration. Hb levels significantly increased after three months of treatment, which was associated with an increase in SCFA-producing bacteria in fecal samples, a decrease in inflammatory mediators, and an improvement in iron utilization. They concluded that these effects were at least partly mediated by an improved diversity and abundance of SCFA-producing intestinal bacteria, probably via the activation of HIF [181]. More recently, Roxadustat has been shown to improve gut barrier function in Caco2 cells treated with the uremic toxin homocysteine and the damage of the colonic epithelium in CKD rats [182]. Finally, FG-4592 (Roxadustat) has been found to relieve diabetic kidney disease (DKD) severity in anemic patients and DKD mice, an effect mediated by changes in the gut microbiota by upregulating the production of beneficial gut-derived metabolites [183].

## 11. SGLT2i and Anemia: Possible Role of Gut Microbiota

Sodium-glucose cotransporter 2 inhibitors (SGLT2is) have demonstrated cardiovascular and renal benefits in patients with type 2 diabetes mellitus, heart failure, or CKD. Since the first studies, an initial increase in Hb/hematocrit levels was observed due to several mechanisms, including hemoconcentration, increased EPO levels and erythropoiesis, improved iron metabolism, or reduced inflammation [184]. Furthermore, mediation studies found that the increase in Hb was strongly associated with the cardiorenal benefits of these drugs [184]. More recently, SGLT2is have shown to modulate the gut microbiome, with a lower relative abundance of bacteria taxa capable of fermenting phenylalanine and tryptophan to uremic toxins, resulting in lower plasma levels of these compounds (including PCS), which may be an additional beneficial mechanism of these drugs in anemia [185].

In CKD mice, the administration of canagliflozin decreased the plasma levels of IS and PCS and increased cecal SCFAs. This was associated with an improved composition of microbiota [186]. In type 2 diabetic mice, dapagliflozin treatment modified intestinal microbiota, increasing Ruminococcaceae and proteobacteria, whereas metformin enhanced Ruminococcaceae, Muribaculaceae, Lactobacillaceae, and Bifidobacteriaceae. These findings suggest that dapagliflozin and metformin produced complementary effects on the main beneficial gut bacteria [187]. The combination of dapagliflozin and sodium butyrate in db/db mice decreased abdominal fat and induced changes in intestinal microbiota [188]. Dapagliflozin administered to diabetic rats improved arterial dysfunction by decreasing arterial stiffness, reducing inflammatory markers, and altering microbiota composition [189]. Phlorizin, the precursor of glifozins, was also found to positively modulate gut microbiota [190]. Finally, the combination of dulaglutide and empagliflozin in a non-diabetic mouse model of non-alcoholic steatohepatitis (NASH) induced significant anti-inflammatory effects by modulating the proinflammatory immune response and microbiome dysbiosis [191]. These data suggest that the increases in hemoglobin associated with SGLT2is may be mediated in part by improving the gut microbiota.

## 12. AST-120

The oral adsorbent AST-120, in addition to the adsorption of the uremic toxin precursors in the gut, modulates gut microbiota in animal models, thereby influencing its metabolomic profiling [192,193]. Furthermore, AST-120 is associated with attenuated damage to the epithelial tight junction in the colon, reduced plasma endotoxin levels, and markers of inflammation and oxidative stress in CKD rats [193,194].

Recent research was conducted in CKD patients to assess the effects of AST-120 on the gut microbiota and related metabolomic profiling in patients with advanced CKD. Thirty-two CKD patients (stage 4–5) and 24 non-CKD controls were enrolled. The CKD patients exhibited a significant gut dysbiosis and variations in serum fatty acid levels. The administration of AST-120 was associated with changes in the gut microbiota composition, specifically increasing fatty acid-producing bacteria, together with shifts in microbial gene enrichment of fatty acid biosynthesis and changes in serum SCFA/medium-chain fatty acids, while the abundance of microorganisms responsible for inflammation-related gastrointestinal diseases, metabolic disorders, and uremic toxins were decreased. These results suggest that AST-120 administration ameliorate uremia-induced gut dysbiosis with changes in its byproducts [195]. However, in this study, no apparent differences in Hb levels were observed between CKD patients receiving or not receiving AST-120 (9.54 ± 1.71 vs. 9.35 ± 1.37 g/dL, respectively). Furthermore, AST-120 administration can reduce uremic toxins, systemic oxidative stress, and inflammation [196]. However, additional studies on the effect of AST-120 in delaying/improving anemia in CKD are needed.

## 13. Conclusions

There is evidence that the dysbiotic gut microbiota is involved in the anemia in CKD patients though different mechanisms, including the accumulation of uremic toxins, increased inflammation, and the reduced production of SCFAs. Several strategies may improve the gut dysbiosis of renal patients, including the dietary control of phosphate to avoid phosphate binders, the use of intravenous iron instead of oral iron, the concomitant use of probiotics when antibiotics are necessary, a better management of volume in HD patients and CKD patients with heart failure, and the prescription of healthier DF-rich diets with prebiotic effects. Roxadustat could partly improve ESA hyporesponsiveness by modifying gut bacteria in addition to its recognized effects on erythropoiesis and iron metabolism. Similarly, SGLT2is have shown to increase Hb levels in CKD patients, which may be in part due to its beneficial effects on gut microbiota and uremic toxin reduction. More studies are needed to further elucidate the complex interactions between gut microbiota and renal anemia and to develop effective microbiota-targeted therapies for the treatment of renal anemia.

## Figures and Tables

**Table 1:** Mechanisms related to gut dysbiosis involved in anemia in CKD.

Mechanism	Consequence
**1.** **Increased uremic toxins**	
- Indoxyl sulfate	Induces eryptosis Inhibits EPO synthesis Inhibits erythropoiesis Induces hepcidin synthesis
- Polyamines	Decrease proliferation and maturation of CFU-E
- ADMA	Suppression of EPO-R expression
- P-cresol and TMAO	Impair gut permeability
**2.** **Inflammation**	
- Bacteria-derived LPS	Reduces EPO expression
- Cytokines	Suppress erythropoiesis Induce erythrophagocytosis Impair iron metabolism/Increase hepcidin levels
**3.** **Reduced production of SCFA**	Impairs gut permeability Enhances inflammation Enhances oxidative stress Increases uremic toxin production

## Data Availability

No new data were created or analyzed in this study.
